# DksA-Dependent Transcriptional Regulation in *Salmonella* Experiencing Nitrosative Stress

**DOI:** 10.3389/fmicb.2016.00444

**Published:** 2016-03-31

**Authors:** Matthew A. Crawford, Calvin A. Henard, Timothy Tapscott, Steffen Porwollik, Michael McClelland, Andrés Vázquez-Torres

**Affiliations:** ^1^Department of Immunology and Microbiology, University of Colorado School of MedicineAurora, CO, USA; ^2^Molecular Biology Program, University of Colorado School of MedicineAurora, CO, USA; ^3^Department of Pathology and Laboratory Medicine, University of California, IrvineIrvine, CA, USA; ^4^Veterans Affairs Eastern Colorado Health Care SystemDenver, CO, USA

**Keywords:** DksA, *Salmonella*, nitric oxide, reactive nitrogen species (RNS), transcriptional regulation, metabolism, cysteine

## Abstract

Redox-based signaling is fundamental to the capacity of bacteria to sense, and respond to, nitrosative and oxidative stress encountered in natural and host environments. The conserved RNA polymerase regulatory protein DksA is a thiol-based sensor of reactive nitrogen and oxygen species. DksA-dependent transcriptional control promotes antinitrosative and antioxidative defenses that contribute to *Salmonella* pathogenesis. The specific adaptive changes mediated by DksA in response to reactive species, however, have not been elucidated. Herein, we characterize DksA-dependent changes in gene expression in *Salmonella enterica* experiencing nitrosative stress. Genome-wide expression analysis of wild-type and Δ*dksA Salmonella* exposed to the nitric oxide (^•^NO) donor DETA NONOate demonstrated ^•^NO- and DksA-dependent regulatory control of 427 target genes. Transcriptional changes centered primarily on genes encoding aspects of cellular metabolism. Several antioxidants and oxidoreductases important in redox buffering, ^•^NO detoxification, and damage repair were also observed to be up-regulated in an ^•^NO- and DksA-dependent manner. Compared to wild-type bacteria, ^•^NO-treated Δ*dksA Salmonella* exhibited a de-repression of genes encoding components of iron homeostasis and failed to activate sulfur assimilation and cysteine biosynthetic operons. As cysteine is integral to efficient antinitrosative and antioxidative defense and repair programs, we further examined the redox-responsive transcriptional control of cysteine biosynthesis by DksA. These investigations revealed that the activation of genes comprising cysteine biosynthesis also occurs in response to hydrogen peroxide, is dependent upon the redox-sensing zinc finger motif of DksA, and requires the transcriptional regulator CysB. Our observations demonstrate that DksA mediates global adaptation to nitrosative stress in *Salmonella* and provide unique insight into a novel regulatory mechanism by which cysteine biosynthesis is controlled in response to reactive oxygen and nitrogen species.

## Introduction

The generation of nitric oxide (^•^NO) by phagocytes is a central component of mammalian host defense against pathogenic bacteria (Fang, 1997). Produced enzymatically in response to pro-inflammatory stimuli via the inducible nitric oxide synthase (iNOS), ^•^NO exerts antimicrobial effects by chemically modifying biomolecules in the microbial cell (Stuehr, 1999; Henard and Vazquez-Torres, 2011). [Fe-S] clusters and redox-sensitive cysteine thiols in the catalytic sites of a number of enzymes involved in intermediary metabolism are among the principal targets of ^•^NO and derived reactive nitrogen species (RNS; Henard and Vazquez-Torres, 2011; Richardson et al., 2011). Nitrosative modification of enzymes such as aconitase and dihydroxyacid dehydratase, constituents of the tricarboxylic acid cycle and branched-chain amino acid biosynthesis respectively, imposes considerable metabolic restriction that contributes to the bacteriostatic effects of nitrosative stress.

In response to RNS, bacteria induce the expression of protective detoxification, repair, and metabolic programs (Antelmann and Helmann, 2011). These adaptive changes are typically controlled by transcriptional regulators that contain an RNS-modifiable metal or thiol center which, upon nitrosative modification, facilitates changes in regulatory function and induces antinitrosative defenses. Investigations by our laboratory have identified the RNA polymerase (RNAP) regulatory protein DksA as a thiol-based sensor of RNS and reactive oxygen species (ROS) important to the ability of *Salmonella* to resist nitrosative and oxidative stress encountered in host environments (Henard et al., 2014).

DksA is a highly conserved transcriptional regulator that, often together with the nucleotide alarmone guanosine tetraphosphate (ppGpp), directs metabolic adaptations to nutrient limitation collectively known as the stringent response (Dalebroux and Swanson, 2012). Following exposure to RNS, thiol groups in the four-cysteine zinc finger motif of *Salmonella* DksA incur nitrosative modifications that trigger a loss of protein α-helicity and release of Zn^2+^ (Henard et al., 2014). These changes are associated with altered transcriptional control by DksA, indicating that this RNAP regulatory protein may function to counter the metabolic restrictions imposed by nitrosative stress. Indeed, Δ*dksA Salmonella* are hyper-susceptible to the bacteriostatic effects of ^•^NO and are also attenuated in macrophage and murine models of infection (Henard et al., 2010; Henard and Vazquez-Torres, 2012).

Although DksA-dependent transcriptional changes have been reported in *Salmonella* experiencing nitrosative stress (Henard et al., 2010, 2014), global changes in gene expression have not been examined. Here we have used a comparative transcriptomic study to identify ^•^NO- and DksA-dependent changes in gene expression in *Salmonella*. Our investigations highlight the importance of metabolic adaptation in limiting the deleterious effects of reactive species and better define the specific contributions of DksA to antinitrosative defense.

## Materials and methods

### Bacterial strains and growth conditions

*Salmonella enterica* serovar Typhimurium strain 14028s and its derivatives used in this study are presented in Table 1. Bacterial cultures were grown overnight in Luria-Bertani (LB) medium at 37°C with continuous shaking. *In vivo* transcriptional responses were examined following subculture in minimal E salts glucose (EG) medium (1.7 mM MgSO_4_, 9.5 mM citric acid, 57.4 mM K_2_HPO_4_, 16.7 mM H_5_NNaPO_4_, and 22.2 mM glucose) containing 0.1% casamino acids (EGCA medium) and supplemented with 10 μM FeCl_3_ and 2 μg/ml thiamine.

**Table 1:** **Bacterial strains used in this study**.

**Strain**	**Genotype**	**Source**
*Salmonella enterica* serovar Typhimurium strain 14028s	wild-type	ATCC
AV09294	Δ*dksA*::FRT	Henard et al., 2010
AV10359	Δ*dksA*::FRT put::*dksA*::Cm	Henard and Vazquez-Torres, 2012
AV08261	Δ*dksA*::Cm Δ*relA*::FRT Δ*spoT*::FRT	Henard and Vazquez-Torres, 2012
AV10360	Δ*dksA*::FRT put::*dksA* C114S::Cm	Henard and Vazquez-Torres, 2012
AV11304	wild-type pQF50::P*cysD*	This study
AV11305	Δ*dksA*::FRT pQF50::P*cysD*	This study
AV11205	Δ*dksA*::FRT put::*dksA*::Cm pQF50::P*cysD*	This study
AV11206	Δ*dksA*::FRT put::*dksA* C114S::Cm pQF50::P*cysD*	This study
AV11063	Δ*cysB*::FRT pQF50::P*cysD*	This study

### Genetic manipulations

The disruption of *dksA* and *relA*/*spoT* in *Salmonella*, as well as complementation of Δ*dksA* bacteria with native or C114S *dksA* alleles has been reported previously by our laboratory (Henard et al., 2010, 2014). The deletion of *cysB* was accomplished using the method of Datsenko and Wanner with minor modification (Datsenko and Wanner, 2000). Briefly, PCR amplicons containing a chloramphenicol resistance cassette flanked by the flippase recognition target (FRT) were generated from pKD3 using primers Δ*cysB* F and R (Table 2) that contain homology to *cysB*. Purified products were electroporated into *Salmonella* harboring the plasmid pTP223 (tetracycline resistant) that encodes an isopropyl β-D-1-thiogalactopyranoside (IPTG)-inducible λ Red homologous recombinase system. Transformants were passaged in the absence of tetracycline for spontaneous loss of pTP233 and the chloramphenicol cassette was removed via the introduction of the temperature sensitive plasmid pCP20 expressing Flp recombinase that recombines flaking FRT sites. The removal of pCP20 was accomplished by passage at 37°C. In-frame deletion of *cysB* was sequence verified.

**Table 2:** **Primers used in this study**.

	**Primer sequence (5′ → 3′)**
**CLONING**	
Δ*cysB*	F: ATAAACGATGGCCTGATGGCGCTAATCTGGATGATGTATTGCTGGAGCTGCTTCGAAGTT
	R: TTTGTCTGCCATGCCACTACGACACAAACCGACGGTGATAATATGAATATCCTCCTTA
**qPCR**	
*rpoD*	F: GTGGCTTGCAATTCCTTGAT
	R: AGCATCTGGCGAGAAATA
	Probe: 6-FAM-ATAAGTTCGAATACCGTCGCG-3BHQ-1
*feoB*	F: GCCGAAAATATTCAGGACGA
	R: CTGCCGAATTTTTGATCCAT
	Probe: 6-FAM-ATCGAAGCAAGTAAAGGCGA-3BHQ-1
*sitA*	F: CTCCTGATTGCCGGTATTGT
	R: CCCTGCGCTCGTTTAATATC
	Probe: 6-FAM-GCGGAAGTCAGCTCGATTAC-3BHQ-1
*sufA*	F: TTAAGCGTAAAACAGACGGGATG
	R: GCCCTTCCTGTACGTAATCCAC
	Probe: 6-FAM-TCCCGAACGGTATCCAGAACATAGCCA-3BHQ-1
*cysD*	F: TAATCCGGTCATGCTGTACTCCATTGG
	R: GCGAAAGGCGTACATCTCACGGAA
	Probe: 6-FAM-ATCAACGTGCAATAGCGGGAA-3BHQ-1
β**-Galactosidase**	
P*cysD*	F: GCGGGGTACCACCAGGCCCTGGTGTA
	R: GGCCGCAAGCTTGATAATGTGGATAC

### Microarray

Overnight cultures of *Salmonella* were diluted 1:100 in 25 ml of EGCA medium supplemented with 10 μM FeCl_3_ and 2 μg/ml thiamine, grown to an OD_600_ of 0.4, and treated **±** 5 mM of the ^•^NO donor DETA NONOate (dNO; Cayman Chemical Company. Ann Arbor, Michigan) for 30 min at 37°C with continuous shaking. This concentration of dNO produces a continuous flux of 5 μM ^•^NO (Henard and Vazquez-Torres, 2012). Cultures were mixed 5:1 with an ice-cold mixture of phenol (5%)/ethanol (95%) and incubated on ice for 20 min prior to RNA isolation using the High Pure RNA Isolation Kit (Roche. Basel, Switzerland) according to the manufacturer's instructions for bacterial RNA extraction. DNA was removed from RNA samples by treatment with Turbo DNase (Life Technologies. Carlsbad, California) followed by RNeasy clean-up (Qiagen. Valencia, California). Total RNA (30 μg) was used to generate labeled cDNA using 1200 U Superscript II reverse transcriptase (Life Technologies), 40 ng/μl random hexamer primers, 4 nmol Cy dUTP (GE Healthcare. Buckinghamshire, United Kingdom), and 80 U RNase inhibitor (Roche) in 60 μl reactions. Reverse transcription reactions were incubated for 2 h at 42°C. Labeled cDNA was purified using the QIAquick PCR purification kit (Qiagen).

Labeled cDNA from three biological replicates was hybridized onto a custom Nimblegen tiling array based on the genome of *S*. *enterica* serovar Typhimurium 14028s and containing 384,879 *Salmonella*-specific 46–50 mer oligo probes in 12 nucleotide intervals from both chromosomal strands. Arrays were scanned using the GenePix4000B scanner (Molecular Devices. Sunnyvale, California) with Acuity 4.0 software. Fluorescence was quantified using NimbleScan 2.4 software (Roche). Data were analyzed with WebarrayDB using median within-array and quantile between-array normalizations. Custom algorithms were used to condense data for presentation at open reading frame resolution. All array data have been deposited with the Gene Expression Omnibus under the accession number GSE33529.

### Microarray data visualization

Functional annotation was determined by Gene Ontology relationships using gene enrichment analysis according to the Database for Annotation, Visualization, and Integrated Discovery v6.7 (Huang da et al., 2009). Gene Ontology annotations are defined according to the AmiGO 2 v2.3.2 annotations and ontology toolkit. The volcano plot representation of microarray analysis was generated using annotated genes of *S*. *enterica*. Probes against the same gene among individual replicates were pooled for *p*-value calculation.

### qPCR

cDNA was prepared as described above for microarray analysis from *Salmonella* treated ±5 mM dNO without Cy dUTP labeling. The primer and probe sequences used for qPCR are listed in Table 2. Reactions were prepared using TaqMan Gene Expression Master Mix (Life Technologies) and were incubated at 50°C for 2 min then 95°C for 10 min, prior to 40 cycles of 95°C for 15 s and 57°C for 1 min. Target gene expression was normalized to the house-keeping gene *rpoD* determined for each sample under analogous conditions. The expression of *rpoD* by *Salmonella* has been previously shown to be unaffected by exposure to 5 mM dNO (Henard et al., 2014); this observation is supported by the current microarray data.

### β-galactosidase

The *cysD* promoter region (−300 to +100), inclusive of the CysB binding site, was cloned into the KpnI/HindIII sites upstream of the promoterless *lacZ* gene in plasmid pQF50 using primers P*cysD* F and R (Table 2). Overnight cultures harboring pQF50::P*cysD* were diluted 1:100 in 25 ml of EGCA medium supplemented with 10 μM FeCl_3_ and 2 μg/ml thiamine. After 2 h of growth at 37°C with continuous shaking, bacteria were subcultured 1:10 in fresh medium and grown for an additional 1.5 h before treatment ±5 mM dNO, 750 μM spermine NONOate (sNO; Cayman Chemical Company), or 100 μM hydrogen peroxide (H_2_O_2_) for the indicated amount of time. The expression of *lacZ* transcriptional fusions was quantified spectrophotometrically as β-galactosidase enzymatic activity using the substrate o-nitrophenyl-β-D-galactopyranoside. β-galactosidase activity is expressed in Miller Units as determined using the equation: 1000 × [(OD_420_ – (1.75 × OD_550_))/(T(min) × V(ml) × OD_600_)].

### Statistics

Statistical analysis and graphing were performed using GraphPad Prism 4.0 software; a *p*-value < 0.05 was considered to be significant. Statistically significant differences among treatment groups were determined using one-way analysis of variance with a Bonferroni multiple comparison test or, where appropriate, an unpaired *t*-test. Significance among microarray analyses was determined using an unpaired *t*-test. Genes were considered to be DksA-dependent when expression in response to ^•^NO treatment was significantly different between wild-type and Δ*dksA Salmonella*.

## Results

**Figure 1:**
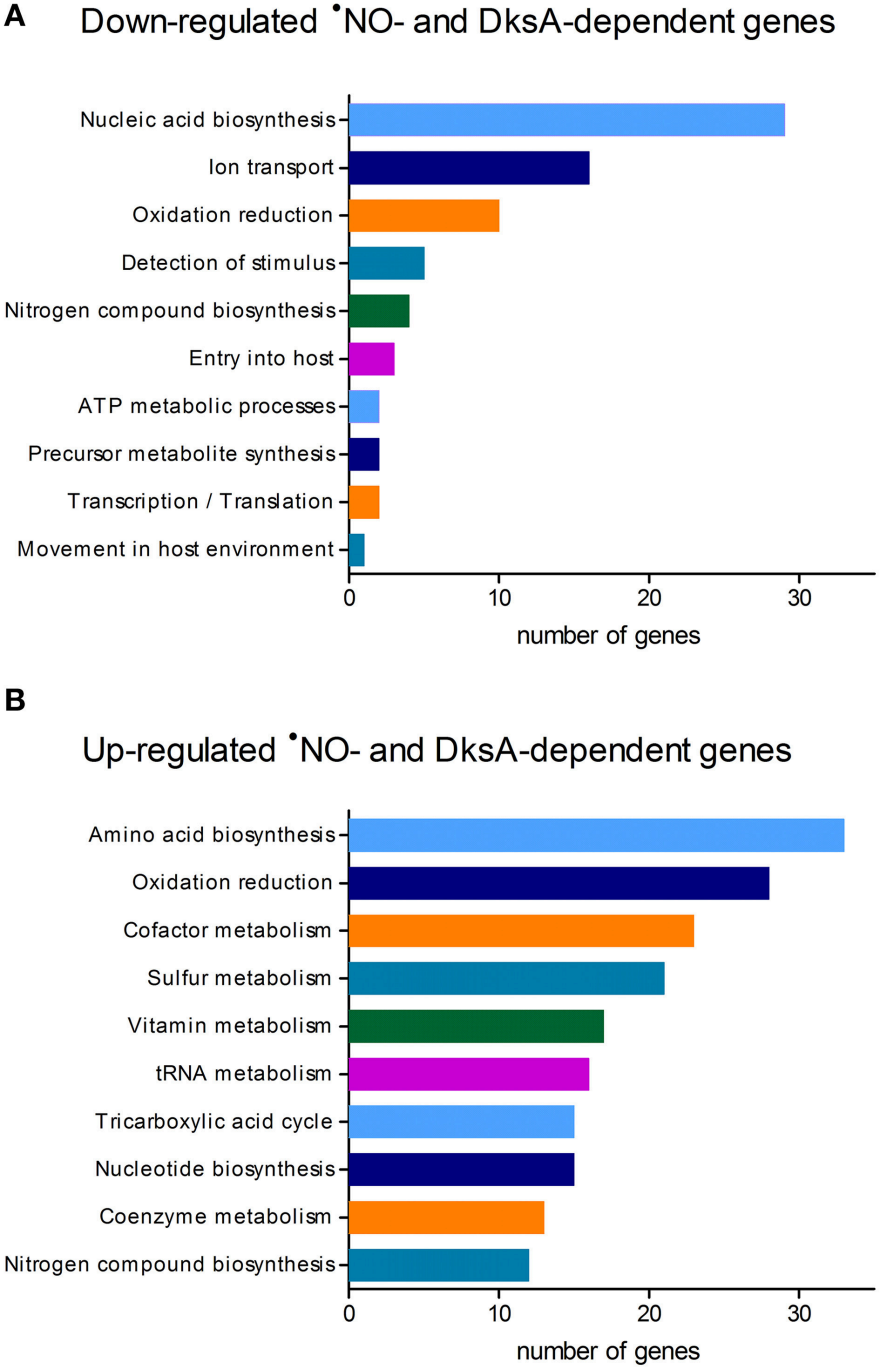
**Functional annotation of ^**•**^NO- and DksA-dependent genes in ***Salmonella*****. Genome-wide expression analysis of wild-type and Δ*dksA S*. *enterica* serovar Typhimurium strain 14028s treated ±5 mM dNO, for 30 min at 37°C with continuous shaking, identified 427 genes (~12% of all annotated open reading frames) as regulated in an ^•^NO- and DksA-dependent manner. Microarrays were performed with cDNA generated from three independent experiments. Distribution of DksA-dependent genes **(A)** down-regulated or **(B)** up-regulated in wild-type *Salmonella* experiencing nitrosative stress; the top 10 categories are shown for each. Functional annotations were determined using Gene Ontology relationships.

To define DksA-mediated adaptive changes in *Salmonella* experiencing nitrosative stress, we compared the transcriptional profiles of wild-type and Δ*dksA Salmonella* following exposure to RNS (Table S1). Genome-wide expression analysis of *Salmonella* treated ±5 mM of the ^•^NO donor dNO identified 427 genes misregulated in Δ*dksA* bacteria as compared to wild-type *Salmonella* (Table S2). Of these ^•^NO- and DksA-dependent loci, 75 were down-regulated and 352 were up-regulated in wild-type bacteria following exposure to dNO. Down-regulated genes included enriched Gene Ontology groups associated with nucleic acid biosynthetic processes and ion transport, in particular iron uptake as exemplified by the *feo* and *sit* operons (Figure 1A). Transcriptional up-regulation by wild-type *Salmonella* in response to dNO was observed among diverse metabolic pathways, most prominently the biosynthesis of amino acids including cysteine, serine, and aromatic amino acids (Figure 1B). Genes encoding enzymes involved in the detoxification of reactive species (e.g., alkyl hydroperoxide reductase and thiol peroxidase) were also up-regulated. Consistent with previous determinations (Henard et al., 2010), DksA was also observed to mediate ^•^NO-responsive regulatory control over components of the pentose phosphate pathway (e.g., glucose-6-phosphate dehydrogenase and gluconate-6-phosphate dehydrogenase), tricarboxylic acid cycle (e.g., succinyl-CoA synthetase and succinic dehydrogenase), glycolysis (e.g., phosphoglycerate mutase), and glutathione (GSH) metabolism (e.g., glutathione synthetase and glutathione reductase).

**Figure 2:**
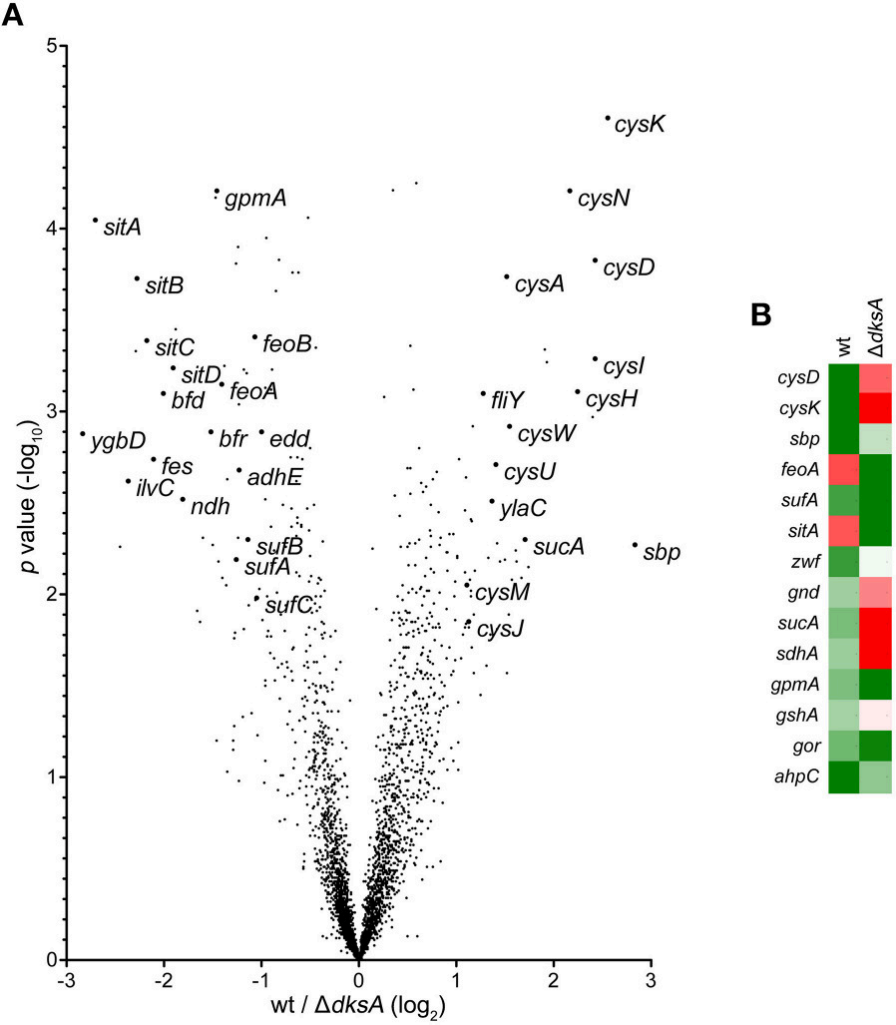
**DksA-dependent transcriptional regulation in ^**•**^NO-treated ***Salmonella*****. **(A)** Positively and negatively regulated loci are represented as a function of the *p*-value using a volcano plot. Expression is depicted as fold change of the ratio of ^•^NO-treated wild-type (wt) and Δ*dksA Salmonella* as compared to control conditions. **(B)** Heat map representing the ratio of selected up-regulated (green) and down-regulated (red) transcripts between ^•^NO-treated and untreated controls.

**Figure 3:**
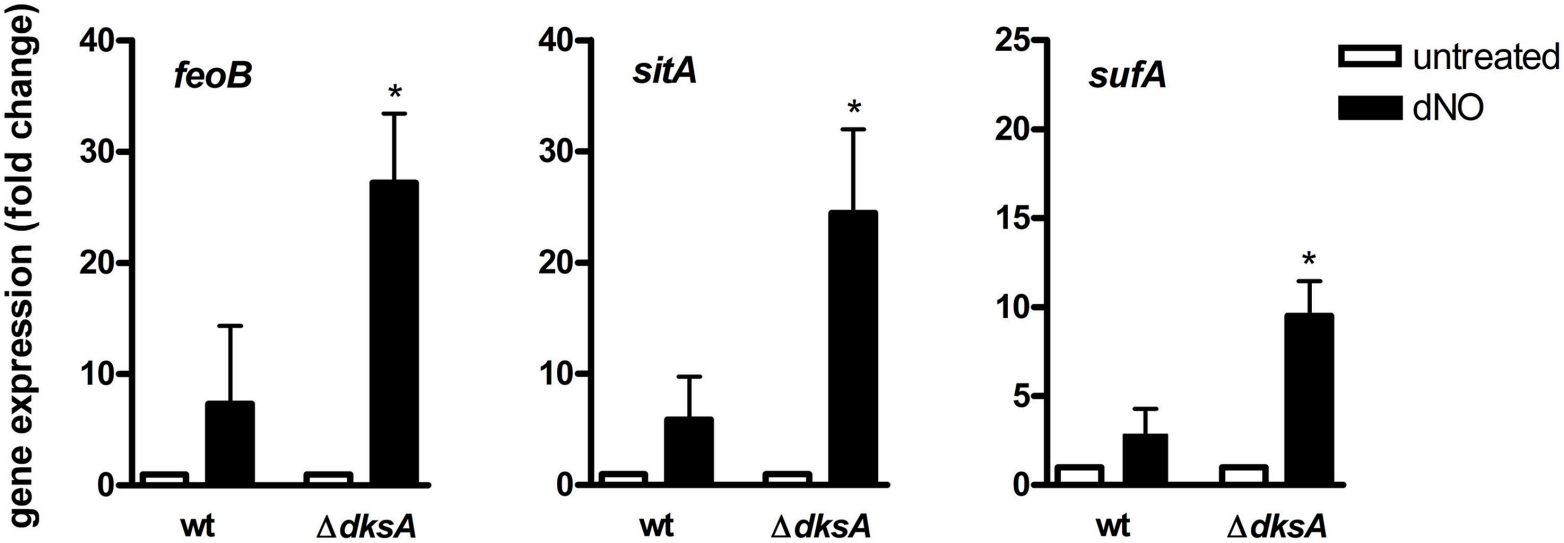
**^**•**^NO- and DksA-dependent transcriptional control of iron homeostasis**. The expression of *feoB, sitA*, and *sufA* in wild-type (wt) and Δ*dksA Salmonella* treated ±5 mM dNO for 30 min was determined using qPCR. Gene expression, normalized to the house-keeping gene *rpoD*, is expressed as fold change compared to the respective untreated control. Data are the mean ± SEM, *n* = 3; ^*^p < 0.05 as compared to dNO-treated wild-type bacteria.

Genes identified as differentially expressed between wild-type and Δ*dksA Salmonella* in response to RNS were further characterized by comparing the ratios of dNO-treated over untreated transcript levels. This analysis is reported as a function of the *p*-value using a volcano plot (Figure 2A). A heat map depicting the specific transcriptional change among selected genes discussed is also presented (Figure 2B). Among the findings highlighted by the preceding analysis was the observation that Δ*dksA Salmonella* experience significant transcriptional de-repression of several genes whose products are involved in iron homeostasis as compared to wild-type bacteria. More specifically, genes encoding the Sit ferrous iron and manganese uptake ABC transporter (*sitABCD*), the Feo ferrous iron importer (*feoAB*), and components of the Suf [Fe-S] cluster assembly and repair system (*sufAB*) were up-regulated in dNO-treated Δ*dksA* bacteria, but generally unchanged in wild-type *Salmonella* upon exposure to dNO. To validate ^•^NO- and DksA-dependent regulatory control in aspects of iron homeostasis, we used quantitative real-time PCR (qPCR) to measure the mRNA levels of *feoB, sitA*, and *sufA* in wild-type and Δ*dksA Salmonella* treated ± dNO (Figure 3). In agreement with the microarray data, this transcriptional analysis demonstrated a significantly greater induction of *feoB, sitA*, and *sufA* in response to RNS in Δ*dksA Salmonella* as compared to analogously treated wild-type bacteria.

**Figure 4:**
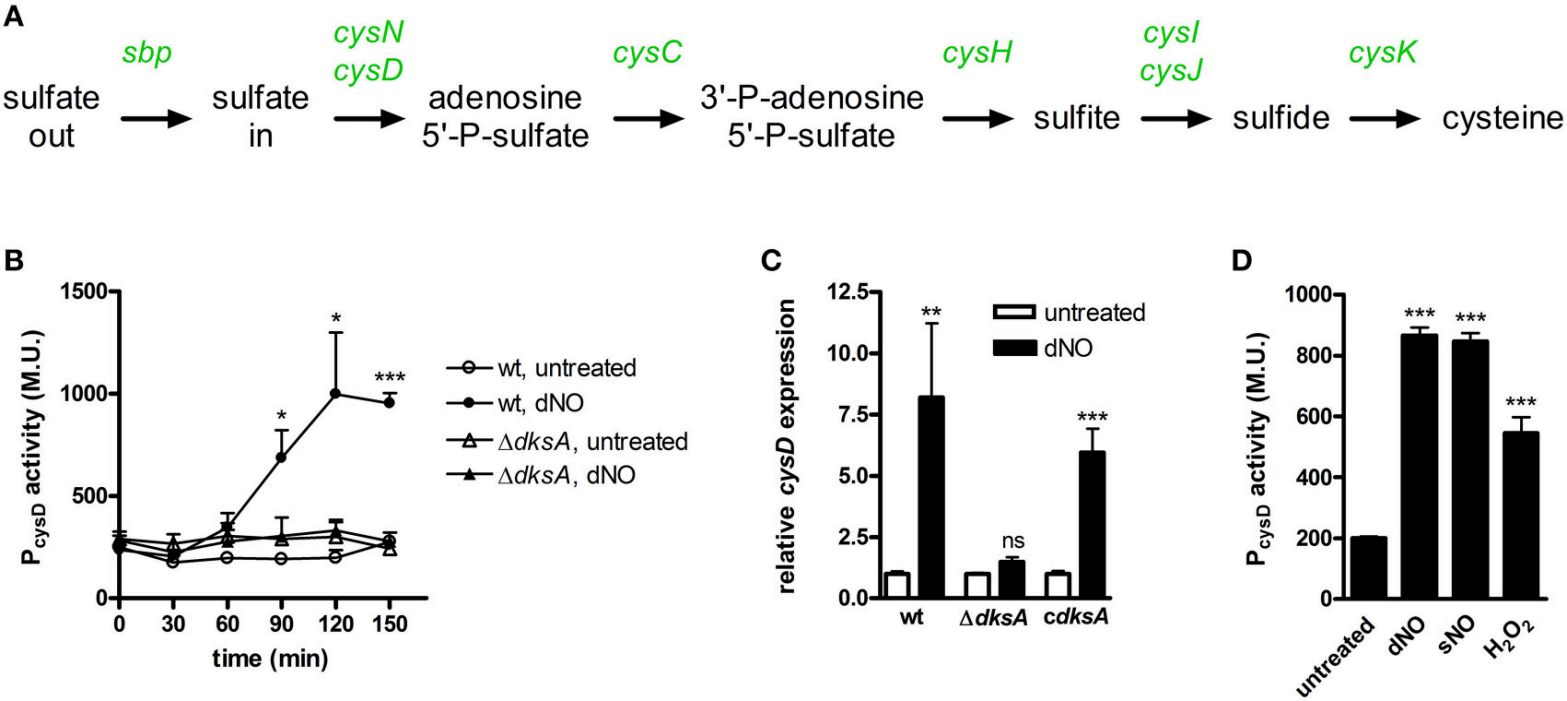
**DksA-dependent transcriptional activation of sulfur assimilation and cysteine biosynthesis in response to nitrosative and oxidative stress. (A)** ^•^NO- and DksA-dependent transcriptional up-regulation of genes encoding sulfur assimilation and cysteine biosynthetic pathways are indicated in green text. **(B)** *cysD* promoter activity in untreated and 5 mM dNO-treated *Salmonella*. Data are expressed as Miller Units (M.U.) and are the mean ± SEM, *n* = 3; ^*^p < 0.05 and ^***^p < 0.001 compared to untreated wild-type *Salmonella*. **(C)** *cysD* expression in wild-type (wt), Δ*dksA*, and *dksA*-null *Salmonella* complemented with the native *dksA* allele (c*dksA*) following treatment ±5 mM dNO for 30 min as measured by qPCR. Gene expression, normalized to the house-keeping gene gene *rpoD*, is expressed as fold change relative to untreated controls. Data are the mean ± SEM, *n* = 3; ^**^p < 0.01, and ns (non-significant) compared to the respective untreated control. **(D)** *cysD* promoter activity 2 h after treatment with 5 mM dNO, 750 μM sNO, or 100 μM H_2_O_2_. Data, expressed as M.U., are the mean ± SEM, *n* = 3; ^***^p < 0.001 compared to untreated *Salmonella*.

Also of particular note among disparate transcriptional responses between wild-type and Δ*dksA Salmonella* was the marked up-regulation of genes that encode proteins involved in sulfur assimilation and cysteine biosynthesis by wild-type bacteria, but not Δ*dksA Salmonella*, in response to dNO. Indeed, the entire pathway for cysteine biosynthesis was observed to be up-regulated in wild-type *Salmonella* exposed to RNS (Figure 4A). To independently test ^•^NO- and DksA-dependent activation of sulfur assimilation and cysteine biosynthesis in *Salmonella* experiencing nitrosative stress, the *cysD* promoter (P*cysD*) was cloned upstream of the promoterless *lacZ* gene in the plasmid pQF50. Consistent with the microarray analysis, wild-type *Salmonella* induced P*cysD*-*lacZ* transcription over time upon exposure to dNO; Δ*dksA* bacteria did not (Figure 4B). Examination of *cysD* expression by qPCR yielded similar results and also demonstrated that complementation of Δ*dksA Salmonella* with the native *dksA* allele restored the induction of *cysD* in these organisms (Figure 4C). We also measured transcriptional changes by wild-type *Salmonella* upon exposure to a second ^•^NO donor, sNO, as well as the ROS H_2_O_2_ (Figure 4D). Exposure of wild-type *Salmonella* to either sNO or H_2_O_2_ induced significant transcriptional activation from P*cysD*.

**Figure 5:**
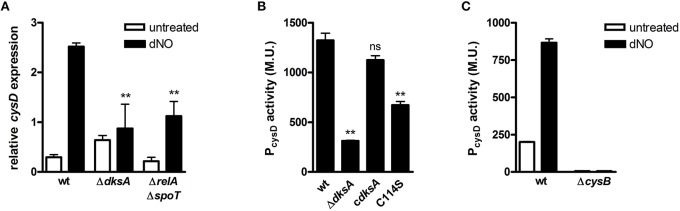
**Cellular requirements for DksA-dependent transcriptional activation of cysteine biosynthesis in response to ^**•**^NO. (A)** *cysD* expression in wild-type (wt), Δ*dksA*, and ppGpp-null (Δ*relA* / Δ*spoT*) *Salmonella* 30 min after treatment ±5 mM dNO as measured by qPCR. Gene expression, normalized to the house-keeping gene *rpoD*, is expressed as fold change relative to untreated controls. Data are the mean ± SEM, *n* = 3; ^**^p < 0.01 and ns (non-significant) compared to dNO-treated wild-type *Salmonella*. **(B)** *cysD* promoter activity in wild-type (wt), Δ*dksA*, and *dksA*-null *Salmonella* complemented with the native (c*dksA*) or C114S *dksA* allele following treatment ±5 mM dNO for 2 h. Data are expressed as Miller Units (M.U.) and are the mean ± SEM, *n* = 3; ^**^p < 0.01 and ns (non-significant) compared to wild-type *Salmonella*. **(C)** *cysD* promoter activity in wild-type (wt) and Δ*cysB Salmonella* 2 h after treatment ±5 mM dNO. Data, expressed as M.U., are the mean ± SEM, *n* = 3.

As cysteine is integral to efficient antinitrosative and antioxidative defense and repair programs, we further examined the redox-responsive transcriptional control of cysteine biosynthesis by DksA. To determine whether the nucleotide alarmone ppGpp participates in the transcriptional activation of cysteine biosynthesis in response to RNS, we examined the induction of *cysD* in ppGpp-null (Δ*relA*/Δ*spoT*) *Salmonella* upon exposure to dNO (Figure 5A). Transcriptional analysis demonstrated ppGpp-null bacteria to activate *cysD* expression in response to nitrosative stress; however, the extent of activation was reduced as compared to wild-type bacteria. Similar analysis demonstrated that disruption of the four-cysteine zinc finger motif of DksA by cysteine to serine point mutagenesis (C114S) significantly reduced the activation of P*cysD* transcription in response to RNS (Figure 5B) and that CysB, a LysR-type transcriptional regulator fundamental to the control of sulfur utilization and cysteine biosynthetic operons (Schell, 1993), is required for ^•^NO- and DksA-dependent activation of P*cysD* in *Salmonella* exposed to RNS (Figure 5C).

## Discussion

Survival within host macrophages is a critical aspect of *Salmonella* pathogenesis and requires that this enteropathogen effectively counter the deleterious effects of phagocyte-derived RNS (Fields et al., 1986; Mastroeni et al., 2000; Vazquez-Torres et al., 2000). Our laboratory has identified the RNAP regulatory protein DksA as a thiol-based sensor of RNS. Thiol groups in the four-cysteine zinc finger motif of DksA become S-nitrosylated in *Salmonella* exposed to acidified nitrite, a primary source of nitrosative stress in the gastric lumen and macrophages (Henard et al., 2014). Nitrosative modification of DksA is associated with the release of coordinated Zn^2+^, as well as changes in protein conformation and regulatory control. We have examined herein global transcriptional changes signaled by DksA in response to nitrosative stress.

Our investigations identified 427 genes as regulated by ^•^NO and DksA in *S. enterica* experiencing nitrosative stress. ^•^NO- and DksA-dependent transcriptional up-regulation was observed among diverse aspects of cellular metabolism and included a number of genes that encode components of central metabolic pathways, in particular the tricarboxylic acid cycle. Aspects of nitrogen metabolism were also up-regulated in wild-type *Salmonella* experiencing nitrosative stress, possibly reflecting a DksA-dependent switch from aerobic to anaerobic respiration in response to RNS-mediated disruptions in the electron transport chain (Husain et al., 2008). The biosynthesis of GSH, an important cellular reductant that adds considerably to the antinitrosative defense of *Salmonella* in an acute model of infection (Song et al., 2013), was also observed to be up-regulated, dependent upon DksA, in wild-type *Salmonella* experiencing nitrosative stress. Genes encoding glutaredoxin-3 and thioredoxin-1 oxidoreductases were similarly up-regulated. Absent from positively regulated, ^•^NO- and DksA-dependent transcripts was *hmpA* which encodes flavohaemoglobin, a prominent ^•^NO detoxifying protein (Poole and Hughes, 2000). In general, our findings are consistent with a previous report which proposed that DksA contributes to antinitrosative defense by amending metabolism to promote the generation of reducing equivalents and biomolecule precursors that are required for the detoxification of reactive species, repair of RNS-mediated damage, and restoration of redox homeostasis (Henard et al., 2010). This notion is similar to the production of NADPH by glucose 6-phosphate dehydrogenase following transcriptional activation by the redox-sensitive SoxRS regulatory cascade in response to oxidative and nitrosative stress (Lundberg et al., 1999).

A number of genes encoding products involved in iron homeostasis and [Fe-S] cluster assembly / repair were found to be up-regulated in ^•^NO-treated Δ*dksA Salmonella*, but generally unchanged in wild-type bacteria. Although nitrosative modification of DksA may relieve transcriptional repression at these loci, this observation could reasonably reflect increased susceptibility by Δ*dksA Salmonella* to RNS-mediated damage of heme and non-heme iron centers in metalloproteins. Thus, the transcriptional up-regulation of iron acquisition and [Fe-S] biogenesis may result from the induction of repair programs independent of DksA. For instance, ^•^NO has been shown to react directly with the Fe^2+^ cofactor of the ferric uptake regulation (Fur) protein, generating a dinitrosyl iron complex that disrupts the ability of Fur to repress target genes that include the *feo, sit*, and *suf* operons (D'Autreaux et al., 2002).

The most apparent disparity between the transcriptional responses of wild-type and Δ*dksA Salmonella* exposed to RNS was the marked up-regulation of cysteine biosynthesis in wild-type bacteria, but not Δ*dksA Salmonella*. As cysteine thiols incur RNS-mediated damage, our investigations indicate that DksA-dependent activation of sulfur assimilation and cysteine production may support the repair and replacement of cysteine-containing proteins. Furthermore, taken together with the observed DksA-dependent transcriptional activation of GSH biosynthesis, increased cysteine production may also be essential for replenishing GSH pools and re-establishing cellular redox balance. Transcriptional activation of *cysD* in wild-type *Salmonella*, but not Δ*dksA* bacteria, in response to nitrosative stress was consistent as measured by microarray, β-galactosidase, and qPCR analyses. However, the extent of *cysD* induction exhibited variability among individual experiments. Although the precise reason(s) for this variation is unknown, it is possible that the physiological state of the bacterial cell in experiments conducted over the course of our investigations may have influenced the degree of up-regulation observed for cysteine biosynthetic operons.

In addition to cysteine, a number of additional amino acid biosynthetic operons were positively regulated by DksA in response to nitrosative stress. These included, among others, genes encoding components of the arginine, serine, glutamate, and aromatic amino acid biosynthetic pathways. These findings indicate that DksA also participates in relieving the metabolic limitations imposed by nitrosative stress. Interestingly, this reinforces the prospect that amino acid shortages arising from nitrosative stress elicit classic stringent control (Hyduke et al., 2007). In support of this notion, *Salmonella* harboring non-functional DksA C114S maintained partial induction of cysteine biosynthesis in response to RNS, and ppGpp contributes to this transcriptional response in *Salmonella*. Although separating thiol-based sensing from canonical stringent control remains to be achieved, it appears that DksA integrates nutritional, oxidative, and nitrosative signals into a coordinated regulatory output that tailors intermediary metabolism to ameliorate stresses encountered by bacteria in their environments.

The activation of amino acid biosynthesis presented here contrasts with previous studies that reported DksA-dependent transcriptional down-regulation of amino acid biosynthesis in response to oxidative and nitrosative stress (Henard et al., 2010, 2014). These disparate findings are likely owing to differences in the levels of nitrosative stress experienced by *Salmonella*. In the current transcriptomic study, changes in gene expression upon exposure to RNS were examined in nutrient-containing medium capable of supporting bacterial strategies that limit RNS-mediated damage. In contrast, previous analyses were performed in phosphate-buffered saline, thus depriving *Salmonella* of nutrients necessary to maintain redox homeostasis and recover from nitrosative insult. Collectively, these distinct observations indicate that DksA may mediate a graded transcriptional response, becoming increasingly down-regulatory as nitrosative stress increases. This type of regulatory mechanism would allow DksA to orchestrate metabolic adaptations that support antinitrosative defense and recovery against manageable levels of nitrosative stress. As defense programs become overwhelmed and nitrosative damage increases, DksA could pivot transcriptional control to conserve cellular resources and promote survival. Indeed, the suppression of amino acid biosynthesis during high levels of nitrosative stress would be expected to promote survival as several essential components of translation, including elongation factor Tu and threonyl-tRNA synthetase, incur nitrosative modifications that result in mistranlastion (Rhee et al., 2005; Wu et al., 2014; Yutthanasirikul et al., 2016).

Cumulatively, thiol-based sensing of RNS by DksA appears to tailor intermediary metabolism to support redox buffering and biomolecule repair, thereby limiting nitrosative damage and promoting *Salmonella* resistance to nitrosative stress.

## Author contributions

All authors contributed substantially to the work reported. MC, CH, SP, MM, and AV designed experimental strategies. MC, CH, TT, and SP performed experiments and analyzed data. MC and TT performed data visualization. MC, CH, and AV wrote the manuscript.

## Funding

This work was supported by the US National Institutes of Health grants R01 AI54959, T32 GM008730, T32 AI052066, and F32 AI08249; the Veterans Administration grant IO1 BX002073; and the Burroughs Wellcome Fund.

### Conflict of interest statement

The authors declare that the research was conducted in the absence of any commercial or financial relationships that could be construed as a potential conflict of interest.
